# Zebrafish as an emerging model to study estrogen receptors in neural development

**DOI:** 10.3389/fendo.2023.1240018

**Published:** 2023-08-17

**Authors:** Marie-José Boueid, Océane El-Hage, Michael Schumacher, Cindy Degerny, Marcel Tawk

**Affiliations:** U1195, Inserm, University Paris-Saclay, Le Kremlin Bicêtre, France

**Keywords:** estrogen (17β-estradiol), estrogen receptor - ESR, GPER, zebrafish, neurogenesis, glia, oligodendrocyte (OL), notch

## Abstract

Estrogens induce several regulatory signals in the nervous system that are mainly mediated through estrogen receptors (ERs). ERs are largely expressed in the nervous system, yet the importance of ERs to neural development has only been elucidated over the last decades. Accumulating evidence shows a fundamental role for estrogens in the development of the central and peripheral nervous systems, hence, the contribution of ERs to neural function is now a growing area of research. The conservation of the structure of the ERs and their response to estrogens make the zebrafish an interesting model to dissect the role of estrogens in the nervous system. In this review, we highlight major findings of ER signaling in embryonic zebrafish neural development and compare the similarities and differences to research in rodents. We also discuss how the recent generation of zebrafish ER mutants, coupled with the availability of several transgenic reporter lines, its amenability to pharmacological studies and *in vivo* live imaging, could help us explore ER function in embryonic neural development.

## Introduction

Estrogens, essentially the three major forms: estrone (E1), estradiol (E2) and estriol (E3), are a group of hormones that are necessary for the development of female characteristics and reproduction (1–4). Estetrol (E4) is also an estrogenic steroid that is exclusively synthesized in the fetal liver during human pregnancy, yet remains with unknown function (5, 6). However, long gone are the days when these hormones were solely considered as “reproductive hormones” since a wealth of data acknowledge estrogens, as well as other reproductive hormones, as essential players in nervous system development and function (7–13). Once secreted, estrogens can be delivered from the periphery into the nervous system *via* the blood stream. Estrogens can also be synthesized locally within the nervous system and target adjacent cells through paracrine activity, or synthetized and signal within the same cells through autocrine activity (14). The very early exposure of vertebrate embryos to estrogens underscores their fundamental role during development. Indeed, the mammalian embryo grows in a rich estrogenic environment, and estrogen is later provided to embryos maternally through the placenta (15). It is also delivered in the egg yolk of oviparous vertebrates (16). Estradiol, being the major female sex hormone and most effective of the three major estrogens, has been the focus of most estrogenic pathway studies in animals and humans.

In all cases, estrogens mainly exert their function *via* interaction with specific receptors, called estrogen receptors (ERs). Estrogens mediate their function *via* classical ERs, or membrane-associated ERs. ERα and ERβ are responsible for genomic estrogen effects, whereby estrogens bind to the ER in the cytoplasm which then dimerizes and translocates to the nucleus, to finally interact with estrogen responsive element (ERE) DNA sequences found in target genes (3). This classical hormone action is defined as slow response mechanism, considering that ERs must shuttle between cytoplasm and nucleus to exert their transcriptional function. However, other studies have reported a very rapid increase in cAMP in response to E2, highlighting a possible interaction with the adenyl cyclase machinery, thus a non-genomic action. This fast non-genomic estrogen activity could be attributed to a specific membrane initiated steroid signal (MISS) on the ER, that allows the latter to translocate to the membrane following posttranslational modifications (3). On the other hand, it was only recently that a 7-transmembrane G protein coupled receptor, GPR30 or GPER (G protein-coupled estrogen receptor), was proposed as a novel non-classical ER that would mediate estrogen rapid signaling (3, 17–21).

The zebrafish is a fantastic vertebrate model to follow highly dynamic activities of neural cells and their interaction with neighboring cells. Its external development makes it an ideal model for genetic manipulation as early as the one-cell stage and provides a vertebrate model for drug screening and signaling analysis. Their ability to absorb drug compounds enables testing of hundreds of molecules in a relatively short time. Furthermore, zebrafish larvae remain transparent throughout the first weeks of development, which enables careful imaging of live cellular and intracellular events, at a level of detail unfeasible in any other vertebrate organism (22–26). Even though zebrafish generation time is similar to rodents, they develop relatively fast when compared to other vertebrate models. Most importantly, they share conserved molecular mechanisms with other organisms, including regulation of neural development (27).

In this review, we will highlight recent findings from zebrafish and rodents that report nuclear and non-genomic activities of ER signaling in embryonic nervous system with a focus on neural development.

## Characterization of estrogen receptors

Even though some hormones vary between humans and animals in their spatial and temporal expression, it is important to note that so far, every animal organism has contributed to our understanding of hormonal function, sometimes with astonishing and unexpected outcomes.

Regarding estrogens, scientists have made a great progress in understanding ligand/receptor interactions, their downstream effectors and contribution to physiological functions. Moreover, additional progress is expected in the coming years to dissect estrogens, and more specifically ER signaling in neural circuit formation and interaction.

Estrogen receptors are part of the so-called nuclear receptors, known for their transcriptional activity by binding to specific response elements. These receptors present a conserved functional domain organization, with four to five shared domains. Among these are i) the N terminal domain that contains the first of two transactivation domains, and is highly variable; ii) the C domain, which contains the highly conserved DNA-binding domain (DBD); and iii) the E domain, which contains the ligand-binding domain (LBD) and the second transactivation domain, that is also well-conserved and responsible for dimerization (28). Indeed, as mentioned above, there are two types of ERs in rodents, ERα and ERβ. Mouse ERα amino acid sequence shares an overall homology of 88.6% and 97.3% with human and rat ERα sequences respectively, while human ERβ shares 89% identity with rat ERβ and 88% with mouse ERβ (29–31). Moreover, rat ERβ shares more than 95% homology in the DBD domain, and 55% amino acid identity in ligand-binding domain with rat ERα (32). Similar findings were observed in mice ERs, whereby the DBD domain presents a high degree of conversation between the two subtypes (96%) (33). Furthermore, whilst ERα and β can form homodimers of either subtype and interact with their response elements, the two ER subtypes are also able to form DNA-binding heterodimers and potentially diversify estrogen signaling pathways (33).

Two types of estrogen receptors are found in zebrafish, Erα and Erβ, encoded by three distinct genes: *erα* or *esr1*, *erβ1* or *esr2b* and *erβ2* or *esr2a*; *erβ* being duplicated. Initial sequence analysis indicated that zebrafish Erα shares 47.1% identity with human ERα, while Erβ1 and β2 had 46.8% and 51.5% identity, respectively, with human ERβ (34, 35). The characterization of these receptors showed Kd values of 0.74 nM for Esr1, 0.75 nM for Esr2a and 0.42 nM for Esr2b (36). Moreover, all ERs were able to induce a reporter gene activity with an ERE that is estrogen dependent. A link between estrogen activity and estrogen responsive element has also been established through a transcriptomic study. This revealed that estrogens stimulate metabolic pathways during zebrafish development, that liver, pancreas and brain are the most responsive organs to estrogen treatment and that estrogen effects on zebrafish development are stage-specific (37).

Apart from the well characterized estrogen nuclear receptors, it has been shown that a G protein coupled receptor, GPR30 or GPER, is activated by E2 at the cell membrane (18). Weigel and colleagues originally isolated and cloned GPR30 from an estrogen receptor (ER)-positive carcinoma cell line (38). They mapped it to chromosome 7p22 and showed that its transcript encodes a 375 amino acid protein. Using SKBR-3 cells, Dong and colleagues found that estrogen binds to GPR30 with a Kd of 2.7 nmol/l (39).

**Figure 1:**
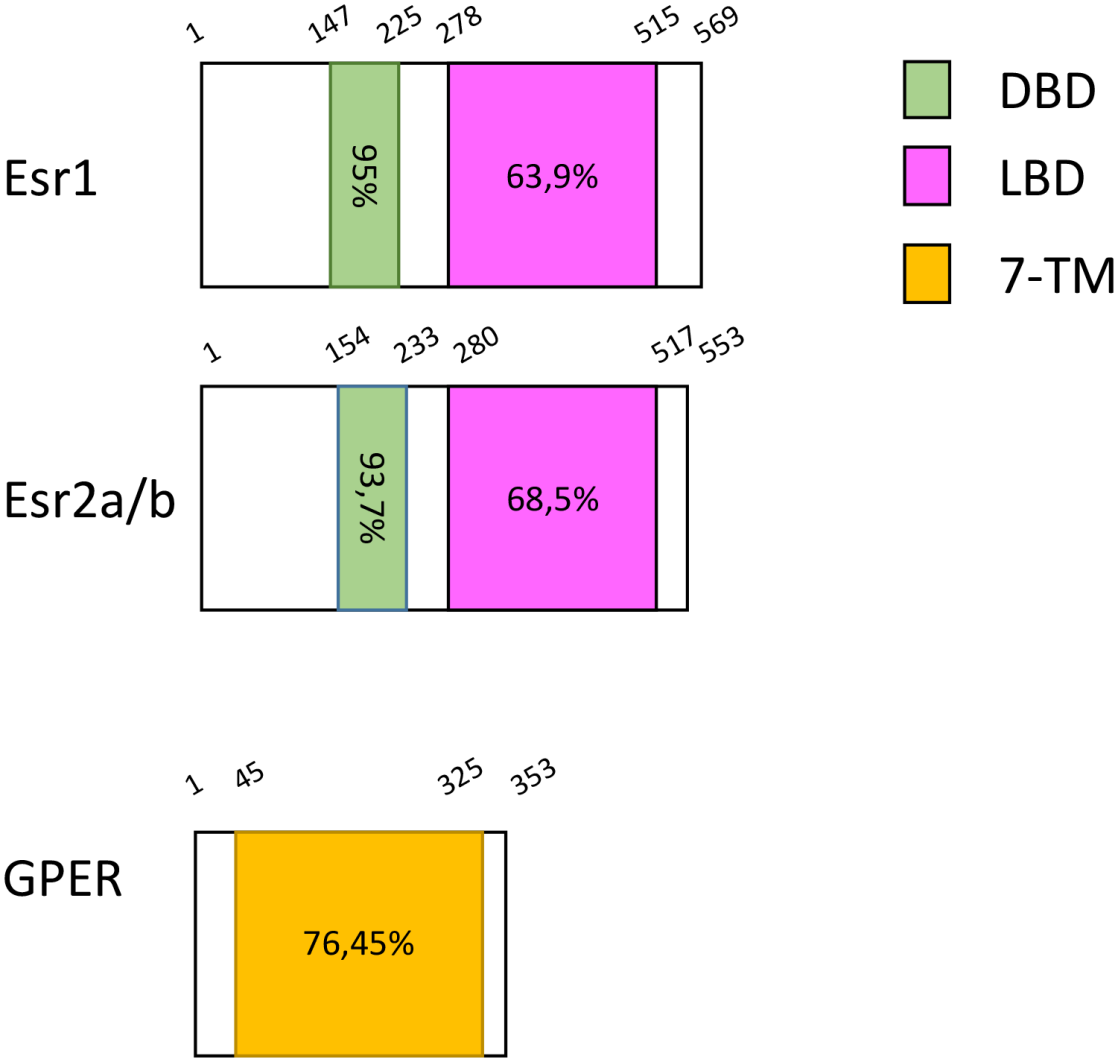
Protein domain architecture of the different zebrafish Estrogen receptors. Length of protein and different domains are highlighted as Amino Acids. Each domain is compared to the equivalent human one and the percentage of identity is shown. DBD, DNA Binding Domain; LBD, Ligand Binding Domain and 7-TM, 7-Transmembrane Domain.

In 2009, Liu and colleagues cloned a full-length cDNA homologous to the GPER of rodents from the testis of zebrafish. It is located on chromosome 3, contains three exons while human ortholog has two; its protein sequence shares 71.5% identity with human GPER (40) (updated sequence analyses are found in **Table 1**, **Figure 1**). Using *gper*-transfected Cos-7 cell line, they revealed the presence of E2-binding sites in GPER, with a Kd of 2.3 nM.

**Table 1:** A comparison of Estrogen receptors’ proteins.

Estrogen receptor	Species	RefSeq	% identity to human
Esr1	Mus muculus	NP_001289460.1	88.98
	Rattus Norvegicus	NP_036821.1	88.17
	Danio rerio	NP_694491.1	57.91
Esr2	Mus muculus	NP_9975590.1	85.77
	Rattus Norvegicus	NP_036886.3	88.68
	Danio rerio (isoform a) Danio rerio (isoform b)	NP_851297.1 NP_777287	56.05 54.7
GPER	Mus muculus	NP_084047.2	86.93
	Rattus Norvegicus	NP_598257.2	86.4
	Danio rerio	NP_001122195.1	71.52

## Expression of estrogen receptors during neural development

The nervous system is a heterogeneous structure of different cell types that originally derive from neural stem cells (NSCs), to give rise to neurons and glia. The terms neurogenesis and gliogenesis are used to define the spatially and temporally controlled transformation of NSCs into differentiated neurons and glia, respectively (41). Thus, the incredible diversity of neurons and glia in the nervous system, results from the tight and fine balance between proliferation and differentiation of neural progenitor cells. This is achieved through the coordination of a multitude of signals, combining extrinsic cues with intrinsic signaling pathways, that are both well defined in time and space. Accordingly, any alteration to the diversity and numbers of neurons or glia, will systematically lead to defects in either brain size, such as microcephaly and macrocephaly, or function, through defective wiring or neural network activity (42).

Estrogen receptors are both widely expressed in the developing fetal rodent brain, from as early as E16.5 for ERα, and E10.5 for ERβ. Erα is more localized to the hypothalamus after birth, while Erβ expression remains more dispersed and found in several areas of the brain and within different cell types, including serotonergic neurons, interneurons, microglia and oligodendrocytes (12).

Zebrafish *erα* is expressed, through different isoforms, from very early stages of development, highlighting a maternal contribution (43). Zygotic expression is also evident, with high levels of expression observed until 96 hours post fertilization (hpf) (latest to be analyzed). The expression of *erβ2* is very low during early stages, but then progressively increases following zygotic transcription. *erβ1* is highly expressed at early stages, drops down and then increases between 24 and 48 hpf (44). However, the highlighted results from qPCR experiments do not correlate with whole mount *in situ* hybridization, since no expression of the three different *er* mRNAs was observed at early stages. *esr1* expression was only detected in the liver at 48 hpf and at 14 days post fertilization (dpf) in the forebrain. *esr2a* and *b* expression is visible at 32 hpf in the forebrain, followed by an expression in the hypothalamus at 48 hpf (34, 44). Thus, the precise spatiotemporal developmental expression of these receptors is yet to be clarified. Work form Olivier Kah’s group shows that estrogens stimulate the expression of aromatase B, a key enzyme responsible for converting androgens to estrogens, in the presence of estrogen receptors, with a higher activity in the presence of Esr2b and a (44). It is possible that fish aromatase is highly expressed in brain regions where ER are strongly expressed too, and that Esr2a and b might be responsible for aromatase expression in radial glial cells in zebrafish brain. Whether there is a direct correlation between the expression and function of ER and aromatase, is yet to be demonstrated, since no functional genetic studies have addressed this issue so far.

GPER expression is mainly studied in adult brain, showing an expression in multiple areas of the central and peripheral nervous systems, including the hypothalamus, spinal cord and dorsal root ganglia (17, 45, 46). Zebrafish *gper*, on the other hand, was found to be expressed at very early stages, and is widely distributed in different regions of the developing brain, as early as 18 hpf (47, 48).

## Role of estrogen receptors in neural development

Most studies have focused on estrogens or molecules and compounds with estrogenic activity as important players in neuroprotection under pathological conditions. Estrogens, indeed, promote neuronal cell survival by increasing the expression of growth factors and/or anti-apoptotic molecules (49, 50). This estrogen activity might also be related to their capacity to modulate dendritic spines, axonal growth, synaptic signaling and plasticity (12, 51–57).

As mentioned above, ERs are widely expressed in the nervous system, however, only a small number of studies have analyzed the impact of estrogen receptor genetic invalidation on neural development *in vivo*. The majority of studies have used selective ER agonists or antagonists to study the role of ERs in biological processes and to demonstrate receptor specificity. To evaluate the effects of estrogen on ERα and ERβ, some have utilized the ERα and ERβ antagonist ICI 182,780 in combination with estrogen (44, 58). However, while ICI 182,780 is an antagonist of ERα and β, it has also been shown to act as an agonist of GPER (59). This suggests that some of the positive effects of estrogen may be mediated by GPER. Researchers have also used selective estrogen receptor modulators (SERMs). SERMs are ER ligands that exhibit preferential binding affinity towards one receptor isotype over the other, and can help clarify the specific contributions of each receptor subtype to the biological effects of estrogen (60). Thus, gene invalidation of each of the ERs remains a good strategy to assess their role(s) in neural development *in vivo.*

Neurogenesis takes place in the two proliferative regions of the mammalian brain, the subventricular zone (SVZ), and the subgranular zone (SGZ) of the dentate gyrus in the hippocampus, where NSCs are abundant (61). Interestingly, both ERα and ERβ, as well as GPER are all expressed in NSCs of rat embryos, highlighting a potential role for these receptors in the behavior of NSCs (8, 62). A wealth of studies shows an important role for estrogens in the proliferation of NSCs. Treating NSCs with E2 enhances the proliferative activity of NSCs, either using human NSCs, or primary cultures of embryonic rat derived NSCs (63). E2 activity, in this case, seems to be predominantly mediated by ERβ (8). Indeed, ERβ**^-/-^** mouse brains show a significant decrease in the number of neurons in the cortex, and their brain is smaller than those of controls (64). Studies from Gustafsson’s lab propose a role for ERβ in neuronal migration and preventing apoptosis during development (65). Using mouse embryonic stem cells (mESCs), studies from the same group found that proliferation was higher and neurogenesis reduced in ERβ KO mESCs. Data provide evidence that ERβ plays an important role in maintaining stem cell identity by curbing proliferation, and possibly favoring nonneuronal fate (13, 66). It remains hard to reconciliate all these data given: i) the important role of E2 in enhancing proliferation of NSCs and stimulating neuronal differentiation *in vivo* and *in vitro*; ii) the smaller brain in ERβ**^-/-^** mice and increased levels of apoptotic neuronal death, while ERβ is shown to mediate apoptosis in neuronal cells; iii) high proliferation in NPCs derived from ERβ KO mice, with no significant difference in apoptosis between controls and KO mice, and no changes in the expression of neuronal markers. Few studies have addressed the role of ERα in neural development, however, some data provide evidence of an important role for ERα in mediating the differentiating and neuroprotective effects of estrogens *in vitro*, in PC12 cells, with a focus on neurite outgrowth (67).

The general consensus, even though results might depend on the timing and location of estrogen activity, is that estrogens stimulate the proliferation of neural stem cells in rodents. Thus, one of the striking differences between zebrafish and rodent studies, is the inhibitory effect of estrogens on cell proliferation in the brain of adult zebrafish, as well as the strong expression of aromatase in radial glial cells (RGs). Using ICI 182,780 as inhibitor of ERs activity (although presenting GPR30 agonist properties), Olivier Kah’s group showed a significant increase in the number of PCNA positive cells in different areas of adult zebrafish brain. Moreover, 17β-estradiol treatment led to a significant decrease in PCNA positive cells, suggesting a role for estrogens in inhibiting cell proliferation through their nuclear receptors, at least partially, in adult zebrafish brain (44, 68–70). As for embryonic studies, treating zebrafish embryos with E2, during nervous system development, decreased the number of BrdU positive cells in the thalamus, olfactory bulbs, telencephalon and preoptic areas, while no difference was observed in mediobasal and caudal hypothalamus (71). Even though some of the areas affected differ between zebrafish adults and larvae, a clear inhibitory effect of estradiol on proliferative activity of neural cells is observed in zebrafish (69, 71). Several studies highlighted a potential role for aromatase in RG development, given its high expression in RGs. However, there is no evidence so far of a role of aromatase in the behavior of RGs, or in neurogenesis *per se*.

While most behavioral studies focused on GPER-selective agonists and antagonists to study the role of GPER in mice behavior (56, 72–74), only few studies assessed its direct role in anxiety and stress responses using GPER KO mice and GPER-deficient rats (75, 76). A potential role for GPER in neural development is yet to be revealed in rodents. A recent study by Pemberton and colleagues has shown, although limited to selective agonist G-1 and E2, a role for GPER activation in neural growth, neural firing activity and intracellular Ca^2+^ rise in primarily cultured E18 rat embryonic neurons (77).

Zebrafish studies have brought more insight into *gper* function during development. Using a morpholino knockdown approach, Lin H. and colleagues revealed an increase in apoptosis and a significant decrease in the expression of some neuronal markers in *gper* morphant embryos, such as Zn-12, Znp-1 and Zn-5 (48). Additional studies, including *gper* KO mutant, are needed for more accurate analysis of *gper* activity in neural development. The first functional analysis of *gper* function during development, using a *gper* KO mutant, is led by Romano and colleagues, in which they show a fundamental role of Gper, centrally, in regulating zebrafish embryonic heart rate, by modulating estrogen and T3 levels in the developing brain (47).

## Estrogen receptors and notch signalling

As mentioned above, estrogens can regulate several aspects of neural development, however, it was not clear until recently how ERs might contribute to neural development. As the Notch pathway is critical to neurogenesis, it was reasonable to consider an interaction between ER and Notch signaling. Both neurogenic genes (*notch*, *delta*), as well as proneural genes (*neurogenin*, *neuroD*), are required for neurogenesis (i.e. neuronal cell fate) in zebrafish and mice. While *notch* is expressed in proliferative neural stem and progenitor cells regions, *neuroD* and *neurogenin* are expressed in postmitotic neurons (78). Thus, the molecular mechanisms driving neuronal development in zebrafish require a similar regulatory cascade to rodents. Moreover, zebrafish present a unique opportunity to analyze the development, behavior and function of not only neurons, but also major glial cell types in the nervous system, from radial glial cells, oligodendrocyte precursor cells, oligodendrocytes, Schwann cells, microglia and the recently identified astrocytes (79–85).

Given the complexity of the nervous system and the diversity of its population during development *in vivo*, a recent study led by Gustafsson’s group tried to address ER and Notch interactions using embryonic stem cells derived from controls and ERβ KO mice. Using a targeted gene-expression profiling in combination with pluripotency markers, the authors provide evidence of reduced neurogenesis and enhanced oligodendrogliogenesis in ERβ KO stem cells, although, there was no significant difference in the expression of neuronal markers. This correlated with higher proliferation, and no measurable differences in apoptosis. Authors also show a sharp decrease (75%) in the expression of *Hes3* transcript in ERβ KO stem cells (66). Indeed, Notch-Hes signaling is a major driver of neural stem cell renewable since it prevents premature differentiation through Notch-Delta lateral inhibition. Hes genes are found highly expressed in neural stem cells and are considered as repressors of neural differentiation. Hence, reducing Hes levels leads to a significant increase in proneural genes’ activity, a premature neurogenesis, as well as rapid depletion of the stem cell pool. This is the first clear demonstration of a role of ERβ in the transcriptional activity of a major signaling player, Notch-Hes, in neurogenesis. This highly defined cell culture system is hence a powerful *in vitro* tool to assess gene-expression profiling. However, the picture is far from clear when it comes to ER activity during nervous system development *in vivo*, where intercellular communication between the different players, and varied extrinsic peripheral and local signaling is established. This added to the complexity of Notch-Hes oscillating activities that drive either proliferation or differentiation *via* other oscillating partners, makes it hard to define a clear role for ER in neurogenesis/gliogenesis (86, 87). Overall, *in vitro* studies have so far established ERβ as a major modulator of Notch-Hes activity in neural stem cells.

Other studies have linked estradiol to dendritogenesis and Notch. Estradiol, by the inhibition of Notch signaling, increases the expression of the proneural gene *neurogenin 3*, and regulates neuritogenesis in developing hippocampal neurons; a mechanism that involves, at least partially, GPER (51). Collectively, data point to a major role of estradiol in mediating several aspects of neural development by modulating Notch signaling, and involving classical ERs, as well as GPER.

With regard to estrogen receptor activity in zebrafish, it has been shown that Esr are fully functional during development. *esrβ2* is shown to regulate the development of sensory hair cells within neuromasts, part of the lateral line organ that mediates directional water movements, prey capture and predator avoidance. The number of sensory hair cells was significantly reduced in *esrβ2* morphants, while supporting cells were present. It is important to note that lateral inhibition is the main mechanism driving zebrafish neuromast differentiation, by imposing a binary fate between hair and supporting cells. Nascent hair cells, expressing Delta protein, inhibit their neighboring cells from adopting hair cell fate, forcing them to become supporting cells; *notch1a* and *notch3* appear to be upregulated in *esrβ2* morphants. Two of notch ligands, *deltaA* and *deltaB* were also upregulated, a mechanism that might explain, at least partially, the suppression of hair cell differentiation (88). On the other hand, it has been shown that *esr1* is required for cell migration within zebrafish posterior lateral line primordium, by repressing chemokine receptor CXCR4 (89). Whether CXCR4 and Notch interact in this particular context is still to be investigated. Moreover, it would be interesting to assess whether this defect is observed in *esr1^-/-^* mutants.

While *in vitro* studies in rodents established a strong link between ERβ and Notch signaling, there remain many open questions: Do ERs contribute to generating the cell diversity within the nervous system of zebrafish? Do they interact with Notch signaling *in vivo*?

## Estrogen receptors and oligodendrogenesis

Estrogen receptors are expressed in both OPCs and oligodendrocytes (OLs), *in vitro* and *in vivo*, suggesting that estrogen signaling may play a role in regulating the proliferation, differentiation, and survival of these cells. Indeed, studies have shown that estrogen treatment can increase the number of oligodendrocytes and myelin production, *in vitro* and *in vivo* (90). In particular, estrogen receptors have been shown to play a role in promoting the differentiation of OPCs into mature oligodendrocytes. Estradiol–ER axis was found to activate the pAkt/mTOR pathway in oligodendrocytes, a pathway known to regulate and promote oligodendrocyte differentiation (72). Studies have suggested that ERα signaling may be particularly important for promoting oligodendrocyte differentiation, while ERβ signaling may be more involved in promoting oligodendrocyte survival and myelin maintenance.

Additionally, well-established animal models of demyelination have shown a prominent role of these nuclear hormone receptors in myelination, by promoting oligodendrocyte maturation and development. It has been suggested that estrogen signaling may have a protective effect on myelin and oligodendrocytes in various conditions that involve demyelination or damage to oligodendrocytes, such as multiple sclerosis. Studies have shown that estrogen treatment can improve myelin repair and reduce inflammation and demyelination in animal models of multiple sclerosis. Mice lacking ERβ in oligodendrocytes are more prone to myelin damage than WT mice in the experimental autoimmune encephalitis model of multiple sclerosis (91). Nevertheless, it was found that ERs are not necessary for SERMs to exhibit their potent effects on OPC differentiation and remyelination *in vivo* (92).

Comparative analysis of the transcriptome in the cortex of ERβ knockout male mice (BERKO) and wild type (WT), revealed upregulation of myelin genes in BERKO mice. Qualitative analysis further demonstrated disrupted layering in the motor cortex of BERKO mice, as evidenced by staining for myelin basic protein (MBP). Transmission electron microscopy (TEM) confirmed a significant increase in axonal myelination thickness in the KO cortex, which was surprising. However, it is possible that loss of ERβ promotes oligodendrogliogenesis, but impairs OL functionality (93). Interestingly, microarray data revealed a significant upregulation of oligodendrocyte-specific factors, including Omg (oligodendrocyte-myelin glycoprotein), and the oligodendrocyte fate-specific transcription factor Olig2 (oligodendrocyte transcription factor 2), in BERKO cultures. Overall, findings suggest that loss of ERβ may enhance oligodendrocyte differentiation and proliferation, possibly through the dysregulation of oligodendrocyte-specific genes (66). Whether ERs have distinct functions during the different stages of OL development, *in vivo*, remains to be clarified.

GPER is expressed in oligodendrocytes within the rat spinal cord and corpus callosum (94). It is also detected throughout the different stages of oligodendrocyte differentiation and promyelinating stages in primary oligodendrocyte cultures. Thus, GPER may play a role in oligodendrocyte development, a function that is yet to be studied.

## Estrogen receptors and neurodevelopmental activity

A recent example of the role of estrogens in the development of zebrafish nervous system comes from Charles Tyler’s lab. In this nicely executed work, authors reveal a new function of estrogens during early brain development. They identify novel estrogen responsive cells, EROB, that play an important role in the development and function of the olfactory sensory system, at least by modulating the intrinsic neuronal activity in the olfactory bulb of developing zebrafish (95). Although, a precise mechanism of estrogen activity within this newly identified glia is still missing, this work identifies a fundamental role of estrogens in the development of the olfactory sensory system. Interestingly, alteration in estrogen activity has also been linked to several neurodevelopmental disorders (96), and estrogenic compounds were able to rescue the nighttime hyperactivity phenotype observed in zebrafish mutant embryos of *contactin associated protein-like 2 (cntnap2)*, an autism-related gene (97). This result might be relevant to understanding the significantly high prevalence of Autism Spectrum Disorder (ASD) in Preterm Infants (98). Indeed, the human fetus is exposed to different levels of estrogens that reach their highest peak during the third trimester, a period characterized with maturation and rapid growth of the brain (99). It is possible that this high prevalence of ASD is related to the reduced hormonal activity, including from estrogens, that preterm infants experience during their development. Studies have shown that increased high risk of ASD is directly linked to loss of placental hormones, particularly in males (100). Even though zebrafish development is substantially different to mammals, notably in the absence of a placenta, it is quite remarkable to observe such a conserved link between hormones, such as estrogens, and autism being established during embryogenesis.

Overall, these studies identify estrogens as modifiers of developmental neural circuits with profound impact on adult behavior. The question remains whether these estrogen related activities signal through ERs.

## Concluding remarks

Neural development describes the process by which neural progenitor cells proliferate, self-renew and generate differentiated cell types in the nervous system, including neurons, oligodendrocytes and astrocytes in a timely manner. Although a wealth of studies provides evidence of a direct role for estrogens in this process, we are just starting to understand the underlying molecular and cellular mechanisms, and the role of different ERs in generating the diversity of neuron/glial cells. Zebrafish offers a unique opportunity to study embryological decisions including neural lineage, the timing of maturation (cells acquiring a certain fate), and the molecular mechanisms of fate decisions. *in vivo* live imaging makes it possible to track individual cells as they divide and differentiate, as well as analyze symmetric and asymmetric divisions that generate differentiating neurons and glial lineages, while renewing the population of progenitor cells. This imaging capability, combined with recently available ER mutants and transgenics that mirror gene expression *in vivo* (e.g. oligos, astrocytes, ERE activity, aromatase activity, notch sensors…) (**Table 2**), will allow us to dissect the role of the different ERs in embryonic neural development and circuit formation. Furthermore, zebrafish mutants will be helpful to address the possible redundancy between the different nuclear and membranous ERs in neural development, an important feature of ER activity that is yet to be tested *in vivo*.

**Table 2:** Available Transgenics/Mutants and Tools to study ERs in zebrafish.

Transgenic/Mutant Lines/Tools	Citation
*gper^-/-^*	(101)
*esr1^-/-^*	(47)
*esr2a^-/-^*	(47)
*esr2b^-/-^*	(47)
*gper* ^-/-^	(47)
*cyp19a1a^-/-^*	(102, 103)
*cyp19a1b^-/-^*	(103)
*Tg(cyp19a1b:GFP)*	(84)
*TgBAC(cyp19a1a:EGFP)*	(102)
*Tg(3ERE-Gal4ff)*	(104)
*Tg(5xERE : GFP)*	(105)
*esr1 MO (5’GGAAGGTTCCTCCAGGGCTTCTCTC3’)*	(89)
*esr1 MO(CATGTAAAACAGGCTGGTCACCTTG)*	(106)
*esr2a MO (AGAGAGTCTTACCTTGTATACTC)*	(106)
*esr2b MO (TTGACCATGAGCATTACCTTGAATG)*	(106)
*gper MO1 (5’TCACATTGGTAGTCTGCTCCTCCAT3’)*	(48)
*gper MO2 (5’AGGTGCTACATACTTCATCTGTGTC3’)*	(48)

## Author contributions

M-JB, OE-H, CD and MT: writing-original draft. M-JB, CD and MT: Figure and tables. All authors contributed to the article and approved the submitted version.

## References

[1] Fuentes N Silveyra P. Estrogen receptor signaling mechanisms. Adv Protein Chem Struct Biol (2019) 116:135–70. doi: 10.1016/bs.apcsb.2019.01.001 PMC6533072 31036290

[2] Arevalo MA Santos-Galindo M Bellini MJ Azcoitia I Garcia-Segura LM. Actions of estrogens on glial cells: Implications for neuroprotection. Biochim Biophys Acta (2010) 1800:1106–12. doi: 10.1016/j.bbagen.2009.10.002 19818384

[3] Arnal JF Lenfant F Metivier R Flouriot G Henrion D Adlanmerini M. Membrane and nuclear estrogen receptor alpha actions: From tissue specificity to medical implications. Physiol Rev (2017) 97:1045–87. doi: 10.1152/physrev.00024.2016 28539435

[4] Bustamante-Barrientos FA Mendez-Ruette M Ortloff A Luz-Crawford P Rivera FJ Figueroa CD. The impact of estrogen and estrogen-like molecules in neurogenesis and neurodegeneration: Beneficial or harmful? Front Cell Neurosci (2021) 15:636176. doi: 10.3389/fncel.2021.636176 33762910 PMC7984366

[5] Holinka CF Diczfalusy E Coelingh Bennink HJ. Estetrol: a unique steroid in human pregnancy. J Steroid Biochem Mol Biol (2008) 110:138–43. doi: 10.1016/j.jsbmb.2008.03.027 18462934

[6] Pinkerton JV Conner EA. Beyond estrogen: advances in tissue selective estrogen complexes and selective estrogen receptor modulators. Climacteric (2019) 22:140–7. doi: 10.1080/13697137.2019.1568403 30895900

[7] Brann DW Lu Y Wang J Zhang Q Thakkar R Sareddy GR. Brain-derived estrogen and neural function. Neurosci Biobehav Rev (2022) 132:793–817. doi: 10.1016/j.neubiorev.2021.11.014 34823913 PMC8816863

[8] Brannvall K Korhonen L Lindholm D. Estrogen-receptor-dependent regulation of neural stem cell proliferation and differentiation. Mol Cell Neurosci (2002) 21:512–20. doi: 10.1006/mcne.2002.1194 12498791

[9] Diotel N Do Rego JL Anglade I Vaillant C Pellegrini E Vaudry H. The brain of teleost fish, a source, and a target of sexual steroids. Front Neurosci (2011) 5:137. doi: 10.3389/fnins.2011.00137 22194715 PMC3242406

[10] Goyette MJ Murray SL Saldanha CJ Holton K Hormones S. Neurosteroids, and glutamatergic neurotransmission: A review of the literature. Neuroendocrinology (2023). doi: 10.1159/000531148 37232008

[11] Maggi A Ciana P Belcredito S Vegeto E. Estrogens in the nervous system: mechanisms and nonreproductive functions. Annu Rev Physiol (2004) 66:291–313. doi: 10.1146/annurev.physiol.66.032802.154945 14977405

[12] McCarthy MM. Estradiol and the developing brain. Physiol Rev (2008) 88:91–124. doi: 10.1152/physrev.00010.2007 18195084 PMC2754262

[13] Nalvarte I Varshney M Inzunza J Gustafsson JA. Estrogen receptor beta and neural development. Vitam Horm (2021) 116:313–26. doi: 10.1016/bs.vh.2021.02.007 33752823

[14] Boon WC Chow JD Simpson ER. The multiple roles of estrogens and the enzyme aromatase. Prog Brain Res (2010) 181:209–32. doi: 10.1016/S0079-6123(08)81012-6 20478440

[15] Bondesson M Hao R Lin CY Williams C Gustafsson JA. Estrogen receptor signaling during vertebrate development. Biochim Biophys Acta (2015) 1849:142–51. doi: 10.1016/j.bbagrm.2014.06.005 PMC4269570 24954179

[16] Radder RS Shine R. Are the phenotypic traits of hatchling lizards affected by maternal allocation of steroid hormones to the egg? Gen Comp Endocrinol (2007) 154:111–9. doi: 10.1016/j.ygcen.2007.05.032 17632106

[17] Arterburn JB Prossnitz ER. G protein-coupled estrogen receptor GPER: Molecular pharmacology and therapeutic applications. Annu Rev Pharmacol Toxicol (2023) 63:295–320. doi: 10.1146/annurev-pharmtox-031122-121944 36662583 PMC10153636

[18] Funakoshi T Yanai A Shinoda K Kawano MM Mizukami Y. G protein-coupled receptor 30 is an estrogen receptor in the plasma membrane. Biochem Biophys Res Commun (2006) 346:904–10. doi: 10.1016/j.bbrc.2006.05.191 16780796

[19] Nilsson S Koehler KF Gustafsson JA. Development of subtype-selective oestrogen receptor-based therapeutics. Nat Rev Drug Discovery (2011) 10:778–92. doi: 10.1038/nrd3551 21921919

[20] Prossnitz ER Hathaway HJ. What have we learned about GPER function in physiology and disease from knockout mice? J Steroid Biochem Mol Biol (2015) 153:114–26. doi: 10.1016/j.jsbmb.2015.06.014 PMC4568147 26189910

[21] Saito K Cui H. Emerging roles of estrogen-related receptors in the brain: Potential interactions with estrogen signaling. Int J Mol Sci (2018) 19(4):1091. doi: 10.3390/ijms19041091 29621182 PMC5979530

[22] Bambino K Chu J. Zebrafish in toxicology and environmental health. Curr Top Dev Biol (2017) 124:331–67. doi: 10.1016/bs.ctdb.2016.10.007 PMC5836480 28335863

[23] Boueid MJ Mikdache A Lesport E Degerny C Tawk M. Rho GTPases signaling in zebrafish development and disease. Cells (2020) 9(12):2634. doi: 10.3390/cells9122634 33302361 PMC7762611

[24] MacRae CA Peterson RT. Zebrafish as tools for drug discovery. Nat Rev Drug Discovery (2015) 14:721–31. doi: 10.1038/nrd4627 26361349

[25] Mrinalini R Tamilanban T Naveen Kumar V Manasa K. Zebrafish - the neurobehavioural model in trend. Neuroscience (2023) 520:95–118. doi: 10.1016/j.neuroscience.2022.12.016 36549602

[26] Nikolaou N Meyer MP. Imaging circuit formation in zebrafish. Dev Neurobiol (2012) 72:346–57. doi: 10.1002/dneu.20874 21309080

[27] Schmidt R Strahle U Scholpp S. Neurogenesis in zebrafish - from embryo to adult. Neural Dev (2013) 8:3. doi: 10.1186/1749-8104-8-3 23433260 PMC3598338

[28] Frigo DE Bondesson M Williams C. Nuclear receptors: from molecular mechanisms to therapeutics. Essays Biochem (2021) 65:847–56. doi: 10.1042/EBC20210020 PMC8628184 34825698

[29] Enmark E Pelto-Huikko M Grandien K Lagercrantz S Lagercrantz J Fried G. Human estrogen receptor beta-gene structure, chromosomal localization, and expression pattern. J Clin Endocrinol Metab (1997) 82:4258–65. doi: 10.1210/jcem.82.12.4470 9398750

[30] Kuiper GG Enmark E Pelto-Huikko M Nilsson S Gustafsson JA. Cloning of a novel receptor expressed in rat prostate and ovary. Proc Natl Acad Sci United States America (1996) 93:5925–30. doi: 10.1073/pnas.93.12.5925 PMC39164 8650195

[31] Tremblay GB Tremblay A Copeland NG Gilbert DJ Jenkins NA Labrie F. Cloning, chromosomal localization, and functional analysis of the murine estrogen receptor beta. Mol Endocrinol (1997) 11:353–65. doi: 10.1210/mend.11.3.9902 9058381

[32] Hanstein B Liu H Yancisin MC Brown M. Functional analysis of a novel estrogen receptor-beta isoform. Mol Endocrinol (1999) 13:129–37. doi: 10.1210/mend.13.1.0234 9892018

[33] Pettersson K Grandien K Kuiper GG Gustafsson JA. Mouse estrogen receptor beta forms estrogen response element-binding heterodimers with estrogen receptor alpha. Mol Endocrinol (1997) 11:1486–96. doi: 10.1210/mend.11.10.9989 9280064

[34] Bardet PL Horard B Robinson-Rechavi M Laudet V Vanacker JM. Characterization of oestrogen receptors in zebrafish (Danio rerio). J Mol Endocrinol (2002) 28:153–63. doi: 10.1677/jme.0.0280153 12063182

[35] Menuet A Pellegrini E Anglade I Blaise O Laudet V Kah O. Molecular characterization of three estrogen receptor forms in zebrafish: binding characteristics, transactivation properties, and tissue distributions. Biol Reprod (2002) 66:1881–92. doi: 10.1095/biolreprod66.6.1881 12021076

[36] Pellegrini E Menuet A Lethimonier C Adrio F Gueguen MM Tascon C. Relationships between aromatase and estrogen receptors in the brain of teleost fish. Gen Comp Endocrinol (2005) 142:60–6. doi: 10.1016/j.ygcen.2004.12.003 15862549

[37] Hao R Bondesson M Singh AV Riu A McCollum CW Knudsen TB. Identification of estrogen target genes during zebrafish embryonic development through transcriptomic analysis. PloS One (2013) 8:e79020. doi: 10.1371/journal.pone.0079020 24223173 PMC3819264

[38] Carmeci C Thompson DA Ring HZ Francke and R.J. Weigel U. Identification of a gene (GPR30) with homology to the G-protein-coupled receptor superfamily associated with estrogen receptor expression in breast cancer. Genomics (1997) 45:607–17. doi: 10.1006/geno.1997.4972 9367686

[39] Thomas P Pang Y Filardo EJ Dong J. Identity of an estrogen membrane receptor coupled to a G protein in human breast cancer cells. Endocrinology (2005) 146:624–32. doi: 10.1210/en.2004-1064 15539556

[40] Liu X Zhu P Sham KW Yuen JM Xie C Zhang Y. Identification of a membrane estrogen receptor in zebrafish with homology to mamMalian GPER and its high expression in early germ cells of the testis. Biol Reprod (2009) 80:1253–61. doi: 10.1095/biolreprod.108.070250 19228597

[41] Mira H Morante J. Neurogenesis from embryo to adult - lessons from flies and mice. Front Cell Dev Biol (2020) 8:533. doi: 10.3389/fcell.2020.00533 32695783 PMC7339912

[42] Silbereis JC Pochareddy S Zhu Y Li M Sestan N. The cellular and molecular landscapes of the developing human central nervous system. Neuron (2016) 89:248–68. doi: 10.1016/j.neuron.2015.12.008 PMC4959909 26796689

[43] Cotter KA Yershov A Novillo A Callard GV. Multiple structurally distinct ERalpha mRNA variants in zebrafish are differentially expressed by tissue type, stage of development and estrogen exposure. Gen Comp Endocrinol (2013) 194:217–29. doi: 10.1016/j.ygcen.2013.09.014 PMC3862120 24090614

[44] Mouriec K Lareyre JJ Tong SK Le Page Y Vaillant C Pellegrini E. Early regulation of brain aromatase (cyp19a1b) by estrogen receptors during zebrafish development. Dev Dyn (2009) 238:2641–51. doi: 10.1002/dvdy.22069 19718764

[45] Hazell GG Yao ST Roper JA Prossnitz ER O'Carroll AM Lolait SJ. Localisation of GPR30, a novel G protein-coupled oestrogen receptor, suggests multiple functions in rodent brain and peripheral tissues. J Endocrinol (2009) 202:223–36. doi: 10.1677/JOE-09-0066 PMC2710976 19420011

[46] Lu H Cui Y Jiang L Ge W. Functional analysis of nuclear estrogen receptors in zebrafish reproduction by genome editing approach. Endocrinology (2017) 158:2292–308. doi: 10.1210/en.2017-00215 28398516

[47] Romano SN Edwards HE Souder JP Ryan KJ Cui X Gorelick DA. G protein-coupled estrogen receptor regulates embryonic heart rate in zebrafish. PloS Genet (2017) 13:e1007069. doi: 10.1371/journal.pgen.1007069 29065151 PMC5669493

[48] Shi Y Liu X Zhu P Li J Sham KW Cheng SH. G-protein-coupled estrogen receptor 1 is involved in brain development during zebrafish (Danio rerio) embryogenesis. Biochem Biophys Res Commun (2013) 435:21–7. doi: 10.1016/j.bbrc.2013.03.130 23583372

[49] Brann DW Dhandapani K Wakade C Mahesh VB Khan MM. Neurotrophic and neuroprotective actions of estrogen: basic mechanisms and clinical implications. Steroids (2007) 72:381–405. doi: 10.1016/j.steroids.2007.02.003 17379265 PMC2048656

[50] Scharfman HE MacLusky NJ. Estrogen and brain-derived neurotrophic factor (BDNF) in hippocampus: complexity of steroid hormone-growth factor interactions in the adult CNS. Front Neuroendocrinol (2006) 27:415–35. doi: 10.1016/j.yfrne.2006.09.004 PMC1778460 17055560

[51] Arevalo MA Ruiz-Palmero I Scerbo MJ Acaz-Fonseca E Cambiasso MJ Garcia-Segura LM. Molecular mechanisms involved in the regulation of neuritogenesis by estradiol: Recent advances. J Steroid Biochem Mol Biol (2012) 131:52–6. doi: 10.1016/j.jsbmb.2011.09.004 21971420

[52] Azcoitia I Barreto GE Garcia-Segura LM. Molecular mechanisms and cellular events involved in the neuroprotective actions of estradiol. Anal sex differences Front Neuroendocrinol (2019) 55:100787. doi: 10.1016/j.yfrne.2019.100787 31513774

[53] Carrer HF Cambiasso MJ Brito V Gorosito S. Neurotrophic factors and estradiol interact to control axogenic growth in hypothalamic neurons. Ann N Y Acad Sci (2003) 1007:306–16. doi: 10.1196/annals.1286.029 14993063

[54] Diotel N Vaillant C Gabbero C Mironov S Fostier A Gueguen MM. Effects of estradiol in adult neurogenesis and brain repair in zebrafish. Horm Behav (2013) 63:193–207. doi: 10.1016/j.yhbeh.2012.04.003 22521210

[55] Frick KM. Molecular mechanisms underlying the memory-enhancing effects of estradiol. Horm Behav (2015) 74:4–18. doi: 10.1016/j.yhbeh.2015.05.001 25960081 PMC4573242

[56] Kim J Szinte JS Boulware MI Frick KM. 17beta-estradiol and agonism of G-protein-coupled estrogen receptor enhance hippocampal memory *via* different cell-signaling mechanisms. J Neurosci (2016) 36:3309–21. doi: 10.1523/JNEUROSCI.0257-15.2016 PMC4792941 26985039

[57] McClure RE Barha CK Galea LA. 17beta-Estradiol, but not estrone, increases the survival and activation of new neurons in the hippocampus in response to spatial memory in adult female rats. Horm Behav (2013) 63:144–57. doi: 10.1016/j.yhbeh.2012.09.011 23063473

[58] Howell A Osborne CK Morris C Wakeling AE. ICI 182,780 (Faslodex): development of a novel, "pure" antiestrogen. Cancer (2000) 89:817–25. doi: 10.1002/1097-0142(20000815)89:4<817::AID-CNCR14>3.0.CO;2-6 10951345

[59] Meyer MR Prossnitz ER Barton M. The G protein-coupled estrogen receptor GPER/GPR30 as a regulator of cardiovascular function. Vascul Pharmacol (2011) 55:17–25. doi: 10.1016/j.vph.2011.06.003 21742056 PMC3216677

[60] Mirkin S Pickar JH. Selective estrogen receptor modulators (SERMs): a review of clinical data. Maturitas (2015) 80:52–7. doi: 10.1016/j.maturitas.2014.10.010 25466304

[61] Urban N Guillemot F. Neurogenesis in the embryonic and adult brain: same regulators, different roles. Front Cell Neurosci (2014) 8:396. doi: 10.3389/fncel.2014.00396 25505873 PMC4245909

[62] Martinez-Cerdeno V Noctor SC Kriegstein AR. Estradiol stimulates progenitor cell division in the ventricular and subventricular zones of the embryonic neocortex. Eur J Neurosci (2006) 24:3475–88. doi: 10.1111/j.1460-9568.2006.05239.x 17229096

[63] Gkikas D Tsampoula M Politis PK. Nuclear receptors in neural stem/progenitor cell homeostasis. Cell Mol Life Sci (2017) 74:4097–120. doi: 10.1007/s00018-017-2571-4 PMC11107725 28638936

[64] Wang L Andersson S Warner M Gustafsson JA. Morphological abnorMalities in the brains of estrogen receptor beta knockout mice. Proc Natl Acad Sci United States America (2001) 98:2792–6. doi: 10.1073/pnas.041617498 PMC30218 11226319

[65] Wang L Andersson S Warner M Gustafsson JA. Estrogen receptor (ER)beta knockout mice reveal a role for ERbeta in migration of cortical neurons in the developing brain. Proc Natl Acad Sci United States America (2003) 100:703–8. doi: 10.1073/pnas.242735799 PMC141060 12515851

[66] Varshney MK Inzunza J Lupu D Ganapathy V Antonson P Ruegg J. Role of estrogen receptor beta in neural differentiation of mouse embryonic stem cells. Proc Natl Acad Sci United States America (2017) 114:E10428–37. doi: 10.1073/pnas.1714094114 PMC5715781 29133394

[67] Merot Y Ferriere F Debroas E Flouriot G Duval D Saligaut C. Estrogen receptor alpha mediates neuronal differentiation and neuroprotection in PC12 cells: critical role of the A/B domain of the receptor. J Mol Endocrinol (2005) 35:257–67. doi: 10.1677/jme.1.01826 16216907

[68] Coumailleau P Pellegrini E Adrio F Diotel N Cano-Nicolau J Nasri A. Aromatase, estrogen receptors and brain development in fish and amphibians. Biochim Biophys Acta (2015) 1849:152–62. doi: 10.1016/j.bbagrm.2014.07.002 25038582

[69] Diotel N Le Page Y Mouriec K Tong SK Pellegrini E Vaillant C. Aromatase in the brain of teleost fish: expression, regulation and putative functions. Front Neuroendocrinol (2010) 31:172–92. doi: 10.1016/j.yfrne.2010.01.003 20116395

[70] Mouriec K Gueguen MM Manuel C Percevault F Thieulant ML Pakdel F. Androgens upregulate cyp19a1b (aromatase B) gene expression in the brain of zebrafish (Danio rerio) through estrogen receptors. Biol Reprod (2009) 80:889–96. doi: 10.1095/biolreprod.108.073643 19129512

[71] Vaillant C Gueguen MM Feat J Charlier TD Coumailleau P Kah O. Neurodevelopmental effects of natural and synthetic ligands of estrogen and progesterone receptors in zebrafish eleutheroembryos. Gen Comp Endocrinol (2020) 288:113345. doi: 10.1016/j.ygcen.2019.113345 31812531

[72] Kumar S Patel R Moore S Crawford DK Suwanna N Mangiardi M. Estrogen receptor beta ligand therapy activates PI3K/Akt/mTOR signaling in oligodendrocytes and promotes remyelination in a mouse model of multiple sclerosis. Neurobiol Dis (2013) 56:131–44. doi: 10.1016/j.nbd.2013.04.005 PMC3674189 23603111

[73] Roque C Mendes-Oliveira J Duarte-Chendo C Baltazar G. The role of G protein-coupled estrogen receptor 1 on neurological disorders. Front Neuroendocrinol (2019) 55:100786. doi: 10.1016/j.yfrne.2019.100786 31513775

[74] Ruiz-Palmero I Hernando M Garcia-Segura LM Arevalo MA. G protein-coupled estrogen receptor is required for the neuritogenic mechanism of 17beta-estradiol in developing hippocampal neurons. Mol Cell Endocrinol (2013) 372:105–15. doi: 10.1016/j.mce.2013.03.018 23545157

[75] Guo L Moon C Niehaus K Zheng Y Ratner N. Rac1 controls Schwann cell myelination through cAMP and NF2/merlin. J Neurosci (2012) 32:17251–61. doi: 10.1523/JNEUROSCI.2461-12.2012 PMC3601465 23197717

[76] Kastenberger I Schwarzer C. GPER1 (GPR30) knockout mice display reduced anxiety and altered stress response in a sex and paradigm dependent manner. Horm Behav (2014) 66:628–36. doi: 10.1016/j.yhbeh.2014.09.001 PMC4213071 25236887

[77] Pemberton K Rosato M Dedert C DeLeon C Arnatt C Xu F. Differential effects of the G-protein-coupled estrogen receptor (GPER) on rat embryonic (E18) hippocampal and cortical neurons. eNeuro (2022) 9(4):ENEURO.0475-21.2022. doi: 10.1523/ENEURO.0475-21.2022 PMC9291730 35788105

[78] Tropepe V Sive HL. Can zebrafish be used as a model to study the neurodevelopmental causes of autism? Genes Brain Behav (2003) 2:268–81. doi: 10.1034/j.1601-183X.2003.00038.x 14606692

[79] Alexandre P Reugels AM Barker D Blanc E Clarke JD. Neurons derive from the more apical daughter in asymmetric divisions in the zebrafish neural tube. Nat Neurosci (2010) 13:673–9. doi: 10.1038/nn.2547 20453852

[80] Chen J Poskanzer KE Freeman MR Monk KR. Live-imaging of astrocyte morphogenesis and function in zebrafish neural circuits. Nat Neurosci (2020) 23:1297–306. doi: 10.1038/s41593-020-0703-x PMC7530038 32895565

[81] Kirby BB Takada N Latimer AJ Shin J Carney TJ Kelsh RN. *In vivo* time-lapse imaging shows dynamic oligodendrocyte progenitor behavior during zebrafish development. Nat Neurosci (2006) 9:1506–11. doi: 10.1038/nn1803 17099706

[82] Park HC Appel B. Delta-Notch signaling regulates oligodendrocyte specification. Development (2003) 130:3747–55. doi: 10.1242/dev.00576 12835391

[83] Sharma K Bisht K Eyo UB. A comparative biology of microglia across species. Front Cell Dev Biol (2021) 9:652748. doi: 10.3389/fcell.2021.652748 33869210 PMC8047420

[84] Tong SK Mouriec K Kuo MW Pellegrini E Gueguen MM Brion F. A cyp19a1b-gfp (aromatase B) transgenic zebrafish line that expresses GFP in radial glial cells. Genesis (2009) 47:67–73. doi: 10.1002/dvg.20459 19101983

[85] Mikdache A Boueid MJ Lesport E Delespierre B Loisel-Duwattez J Degerny C. Timely Schwann cell division drives peripheral myelination in *vivo via* Laminin/cAMP pathway. Development (2022) 149(17):dev200640. doi: 10.1101/2022.02.11.480035 35938454

[86] Imayoshi I Kageyama R. bHLH factors in self-renewal, multipotency, and fate choice of neural progenitor cells. Neuron (2014) 82:9–23. doi: 10.1016/j.neuron.2014.03.018 24698265

[87] Zhang R Engler A Taylor V. Notch: an interactive player in neurogenesis and disease. Cell Tissue Res (2018) 371:73–89. doi: 10.1007/s00441-017-2641-9 28620760

[88] Froehlicher M Liedtke A Groh K Lopez-Schier H Neuhauss SC Segner H. Estrogen receptor subtype beta2 is involved in neuromast development in zebrafish (Danio rerio) larvae. Dev Biol (2009) 330:32–43. doi: 10.1016/j.ydbio.2009.03.005 19289112

[89] Gamba L Cubedo N Ghysen A Lutfalla G Dambly-Chaudiere C. Estrogen receptor ESR1 controls cell migration by repressing chemokine receptor CXCR4 in the zebrafish posterior lateral line system. Proc Natl Acad Sci United States America (2010) 107:6358–63. doi: 10.1073/pnas.0909998107 PMC2851976 20308561

[90] Zorrilla Veloz RI McKenzie T Palacios BE Hu J. Nuclear hormone receptors in demyelinating diseases. J Neuroendocrinol (2022) 34:e13171. doi: 10.1111/jne.13171 35734821 PMC9339486

[91] Khalaj AJ Yoon J Nakai J Winchester Z Moore SM Yoo T. Estrogen receptor (ER) beta expression in oligodendrocytes is required for attenuation of clinical disease by an ERbeta ligand. Proc Natl Acad Sci United States America (2013) 110:19125–30. doi: 10.1073/pnas.1311763110 PMC3839759 24191028

[92] Rankin KA Mei F Kim K Shen YA Mayoral SR Desponts C. Selective estrogen receptor modulators enhance CNS remyelination independent of estrogen receptors. J Neurosci (2019) 39:2184–94. doi: 10.1523/JNEUROSCI.1530-18.2019 PMC6433770 30696729

[93] Varshney MK Yu NY Katayama S Li X Liu T Wu WF. Motor function deficits in the estrogen receptor beta knockout mouse: Role on excitatory neurotransmission and myelination in the motor cortex. Neuroendocrinology (2021) 111:27–44. doi: 10.1159/000506162 31991411

[94] Hirahara Y Matsuda KI Yamada H Saitou A Morisaki S Takanami K. G protein-coupled receptor 30 contributes to improved remyelination after cuprizone-induced demyelination. Glia (2013) 61:420–31. doi: 10.1002/glia.22445 23281138

[95] Takesono A Schirrmacher P Scott A Green JM Lee O Winter MJ. Estrogens regulate early embryonic development of the olfactory sensory system *via* estrogen-responsive glia. Development (2022) 149(1):dev199860. doi: 10.1242/dev.199860 35023540 PMC8881738

[96] Crider A Pillai A. Estrogen signaling as a therapeutic target in neurodevelopmental disorders. J Pharmacol Exp Ther (2017) 360:48–58. doi: 10.1124/jpet.116.237412 27789681 PMC5193073

[97] Hoffman EJ Turner KJ Fernandez JM Cifuentes D Ghosh M Ijaz S. Estrogens suppress a behavioral phenotype in zebrafish mutants of the autism risk gene, CNTNAP2. Neuron (2016) 89:725–33. doi: 10.1016/j.neuron.2015.12.039 PMC4766582 26833134

[98] Agrawal S Rao SC Bulsara MK Patole SK. Prevalence of autism spectrum disorder in preterm infants: A meta-analysis. Pediatrics (2018) 142(3):e20180134. doi: 10.1542/peds.2018-0134 30076190

[99] Schumacher M Liere P Ghoumari A. Progesterone and fetal-neonatal neuroprotection. Best Pract Res Clin Obstet Gynaecol (2020) 69:50–61. doi: 10.1016/j.bpobgyn.2020.09.001 33039311

[100] Vacher CM Lacaille H O'Reilly JJ Salzbank J Bakalar D Sebaoui S. Placental endocrine function shapes cerebellar development and social behavior. Nat Neurosci (2021) 24:1392–401. doi: 10.1038/s41593-021-00896-4 PMC8481124 34400844

[101] Wu XJ Williams MJ Kew KA Converse A Thomas P Zhu Y. Reduced vitellogenesis and female fertility in gper knockout zebrafish. Front Endocrinol (Lausanne) (2021) 12:637691. doi: 10.3389/fendo.2021.637691 33790865 PMC8006473

[102] Dranow DB Hu K Bird AM Lawry ST Adams MT Sanchez A. Bmp15 is an oocyte-produced signal required for maintenance of the adult female sexual phenotype in zebrafish. PloS Genet (2016) 12:e1006323. doi: 10.1371/journal.pgen.1006323 27642754 PMC5028036

[103] Yin Y Tang H Liu Y Chen Y Li G Liu X. Targeted disruption of aromatase reveals dual functions of cyp19a1a during sex differentiation in zebrafish. Endocrinology (2017) 158:3030–41. doi: 10.1210/en.2016-1865 28575219

[104] Lee O Takesono A Tada M Tyler CR Kudoh T. Biosensor zebrafish provide new insights into potential health effects of environmental estrogens. Environ Health Perspect (2012) 120:990–6. doi: 10.1289/ehp.1104433 PMC3404660 22510978

[105] Gorelick DA Halpern ME. Visualization of estrogen receptor transcriptional activation in zebrafish. Endocrinology (2011) 152:2690–703. doi: 10.1210/en.2010-1257 PMC3115614 21540282

[106] Griffin LB January KE Ho KW Cotter KA Callard GV. Morpholino-mediated knockdown of ERalpha, ERbetaa, and ERbetab mRNAs in zebrafish (Danio rerio) embryos reveals differential regulation of estrogen-inducible genes. Endocrinology (2013) 154:4158–69. doi: 10.1210/en.2013-1446 PMC3800766 23928376

